# An Extract from an Article on Salivary Calculus

**Published:** 1851-07

**Authors:** F. Y. Clark


### AN EXTRACT FROM AN ARTICLE ON SALIVARY CALCULUS.

BY F. Y. CLARK, S. D.

Foul breath is very often and very unjustly said to be caused by the stoma h or lungs, but in ninety-nine cases out of a hundred it is caused by the filthy condition of the teeth, which can be very easily proven to the satisfaction of any one, by the simple act of closing the mouth and breathing through the nose. According to my own observation and experience, I might go on and enumerate many other complaints that are caused by the accumulation of this vile concretion; but I think either of the above mentioned enough to induce any one to keep clear of it, if they are clear of it, and if not to have it removed before it gets beyond removing; and the only safe way for you to accomplish this, is by going to a Dentist—to one whom you have confidence in, and have every particle of tartar removed; after which the teeth should be very highly polished, in order to resist foreign substances, that might otherwise collect on them; after having this done you should adopt a systematic course with the brush aided occasionally by some good dentifrice. By closely adhering to the above, you will find that any further accumulation of tartar will be prevented, and those snowy gems of nature will retain their original luster and beauty during the time allotted to man on earth by the great Governor of all powers.

I find there are many in this community who object to the use of the brush, on account, as they say, of its wearing the teeth. But I am well convinced that the brush would not wear the teeth as much in forty years, as uncleanliness would in one. I might go on and name a number of trifling objections of this kind that are urged against this useful and innocent article, but I deem them all as unreasonable as they are absurd, and not worth notice. Again, there are others who dispense with the use of the brush, and substitute something in the shape of a spongy twig with snuff on the end of it. This class I believe is generally known to all by the name of dippers. And of all the modes that ever was invented for the purpose of robbing those human gems of their ivory hue, this is the most successful; for by the use of the twig the snuff is rubbed in between the teeth and between the teeth and gums, where it remains in dark seclusion for weeks and months, encrusting the teeth with the filthiest filth that ever formed within the crimson lips. But the teeth are not the only victims of this black destroyer. After it has been used for a few years to excess, the gums partake of its color, and the breath that once was pure as the gentle breeze of Italy's distant clime, becomes contaminated, and if confined would breed a pestilence more destructive than that of the Asiatic Cholera. And I have often thought that if those fair dippers could but stand over their own breath for two or three hours at a time, as the dentist has often to do, that it would be enough to induce them to lay aside their black bottle and twig, and substitute the brush. But I am often astonished at the recklessness manifested by some in regard to those priceless gems. They do not seem to be aware that in their absence the merry happy smile is dismantled of all its fascinating charms—the power of articulation becomes crippled, and the cheek of youthhood shrunken into old age before they arrive on the stage of maturity. It is enough to cause the stamp of annihilation to be fixed upon every fond hope, every sweet dream that pictures the waste of future bliss.—*Raymond Fencible.*

